# The Mortality Effect of Apparent Temperature: A Multi-City Study in Asia

**DOI:** 10.3390/ijerph18094675

**Published:** 2021-04-28

**Authors:** Ru Cao, Yuxin Wang, Jing Huang, Jie He, Pitakchon Ponsawansong, Jianbo Jin, Zhihu Xu, Teng Yang, Xiaochuan Pan, Tippawan Prapamontol, Guoxing Li

**Affiliations:** 1 Department of Occupational and Environmental Health Sciences, Peking University School of Public Health, 38 Xueyuan Road, Haidian District, Beijing 100191, China; 13051555836@163.com (R.C.); yuxin_wang@bjmu.edu.cn (Y.W.); jing_huang@bjmu.edu.cn (J.H.); 1410307420@bjmu.edu.cn (J.J.); zhihu_xu@sina.com (Z.X.); 1595952019@pku.edu.cn (T.Y.); xcpan@bjmu.edu.cn (X.P.); 2 Peking University School of Nursing, 38 Xueyuan Road, Haidian District, Beijing 100191, China; 1710108506@pku.edu.cn; 3 Environment and Health Research Unit, Research Institute for Health Science, Chiang Mai University, Chiang Mai 50200, Thailand; ponsawansong@gmail.com (P.P.); tippawan.prapamontol@cmu.ac.th (T.P.)

**Keywords:** apparent temperature, mortality, attributable risk, distributed lag nonlinear model

## Abstract

(1) Background: The health effect of temperature has become a rising public health topic. The objective of this study is to assess the association between apparent temperature and non-accidental deaths, and the mortality burden attributed to cold and heat temperature; (2) Methods: The daily data on temperature and deaths were collected from 10 cities in Thailand, Korea and China. We fitted a time-series regression with a distributed lag nonlinear model (DLNM) to derive the health risk of temperature for each city and then pooled them to get the overall cumulative risk by multivariate meta-analysis. Additionally, we calculated the attributable fraction of deaths for heat and cold, which was defined as temperatures above and below minimum-mortality temperature (MMT); (3) Results: There are regional heterogeneities in the minimum mortality percentiles (MMP) and attributable fractions for different countries. The MMP varied from about the 5–10th percentile in Thailand to 63–93rd percentile in China and Korea. The attributable fractions of the total deaths due to short-term exposure to temperature in Asia is 7.62%, of which the cold effect (6.44%) is much higher than the heat effect (1.18%); (4) Conclusions: Our study suggested that apparent temperature was associated with an increase in non-accidental mortality. Most of the temperature-related mortality burden was attributable to cold, except for Thailand.

## 1. Introduction

In the context of global climate-changing [1,2], many epidemiological investigations have focused on the adverse health effects of temperature-related events, such as cold waves, heat waves, and sudden changes in temperature, which are currently highly concerned in the field of environmental health [3,4,5]. In the United States between 1999 and 2009, about 700 people died of heat-related diseases every year, higher than the sum of deaths caused by other environmental events [6]. Chen et al. found the attributable burden of respiratory and cardiovascular disease mortality caused by air temperature in 272 cities in China was 10.57% and 17.48%, respectively [7]. The formulation and implementation of policies and strategies to adapt temperature need further studies which explore the health risk of temperature comprehensively.

A few international studies provided evidence for the association between ambient temperature and adverse health outcomes, including respiratory and circulatory diseases [8,9,10]. However, most research focused on the adverse effects of extreme weather and temperature fluctuations, ignoring the potential health risk caused by the moderate low or high temperature period, which is more frequent [11,12,13,14], leading to the uncomprehensive assessment of the excess morbidity/mortality due to ambient temperature. So it is necessary to model temperature–health dependencies with temperature as a continuous variable and quantify the mortality burden attributable to ambient temperature, including moderate and extreme temperatures. Furthermore, most previous studies [12,15], used relative risk (RR) to quantify the association, providing limited information about the health risk of exposure, without considering the attributable burden. Attributable fraction (AF) is one of the most commonly used methods to quantify the contribution of exposure to adverse health outcomes, defined as the proportion of outcome in the population avoidable if exposure is somehow eliminated [16]. Compared with RR, AF is more suitable for estimating the potential benefits of preventive interventions [17].

The association between ambient temperature and mortality varies across study areas [7,18], which might be due to different regional climates, adaptation responses and susceptibility factors [19]. While these studies were all restricted to a single city or country, large research in multiple countries and regions is needed to achieve reliable results.

To characterize the sensation that humans perceive more objectively, we chose the apparent temperature (AT) to be the exposure metric of temperature, which is a composite biometeorological index combining temperature and relative humidity [20], proposed by Steadman in 1984. It is believed that the biometeorological index can evaluate the health effect of temperature more accurately than typical variable (e.g., average temperature), since the biometeorological index is generally developed based on theoretical stands and combines more than one weather factor [21]. Previous studies also have shown that apparent temperature is more closely related to mortality than other temperature variables [22].

In order to provide scientific evidence for the government’s prevention and control of weather-sensitive diseases, in this study, we aimed to establish the exposure-response relationship between temperature and non-accidental mortality in three countries of Asia: China, Korea and Thailand. Based on the exposure-response relationship, we further evaluate the attributable burden from cold and heat.

## 2. Materials and Methods

### 2.1. Data Source

In this study, time series daily data covering different periods in 10 cities of three Asian countries: China (Ningbo, Tianjin, 2008–2011), Korea (Seoul, Busan, Daegu, Incheon, Gwangju, Daejeon, 2000–2010), Thailand (Bangkok, Chiang Mai, 2013–2016). The data were mainly made up of parallel time series of daily counts of deaths for non-accidental and daily meteorological variables, including temperature and relative humidity. In terms of China, we collected data on the daily number of non-accidental deaths in Ningbo and Tianjin from the Center for Disease Control and Prevention of Ningbo, and the Center for Disease Control and Prevention of Tianjin, respectively, and the meteorological variables were obtained from the China meteorological data service center. Daily deaths due to influenza and air pollution data in Tianjin were collected from the Center for Disease Control and Prevention of Tianjin and the Tianjin Environment Monitoring Center, respectively. Daily mortality and weather data of six cities in Korea between 2000–2010 were collected from a previous multi-city study [23]. With regard to the two Thai cities, we attained the meteorological data from the Thailand Meteorological Department, the Ministry of Digital Economy and Society, and the mortality data from the Strategy and planning division, Ministry of Public Health.

### 2.2. Statistical Analysis

We used a two-stage analysis to evaluate the association between non-accidental mortality and apparent temperature. In the first stage, time series regression was applied to estimate the exposure-response relationship for each city separately. In the second stage, we pooled the estimated exposure-response association of each city using a multivariate meta-analytical model. Finally, to assess the mortality risk of ambient temperature, we calculated the minimum mortality temperature (MMT) and the attributional risk of death due to cold effects and heat effects.

To reflects human somatosensory more objectively, we chose the apparent temperature as an exposure indicator of temperature, which is a comprehensive index of ambient temperature and relative humidity [20]. The apparent temperature is calculated as follows [24] (1):

(1) $$AT=-2.653+0.994*Ta+0.0153*Td^{2}$$

where *Ta* means air temperature and *Td* means dew point temperature.

In the first stage, we applied a Generalized Additive Model (GAM) as the basic framework to establish an exposure-response relationship between apparent temperature and non-accidental mortality for each city. Considering the inherently non-linear relationship, we introduced a distributed lag non-linear model (DLNM) into GAM, which can describe complex non-linear and lagged effects. In order to include the long delay of cold effects and exclude the advanced deaths because of the harvesting effect, we extended the lag period to the 21 days referred to previous literature [25]. The model was as follows (2):

(2) $$\log\left(Y_{t}\right)=\beta Temp_{t,l}+s\left(Trend,df\times year\right)+s\left(RH,4\right)+DOW+a$$

In this model, *Y_t_* represents daily non-accidental death on day *t*; *Temp_t,l_* is cross-basis with DLNM model, the maximum lag time is 21 day; *β* is the coefficient of the cross-basis function of apparent temperature; *s(Trend, df × year)* means cubic B-spline of time with 8 degrees of freedom per year to control for seasonal and long-term trends; *RH* is daily relative humidity; *DOW* means the day of the week effect; *α* is the residual.

In the second stage, we estimated the cumulative risk during the whole lag period and calculated the overall association of the temperature-mortality by pooling city-specific association by meta-analysis. Based on previous studies, climatological and socioeconomic factors could modify the mortality effect of ambient temperature [19]. Considering both the accessibility and comprehensiveness of data from multiple cities, we included average temperature and temperature variation as meta-predictors in the multivariate meta-regression. The heterogeneity was analyzed by the Cochran Q test for qualitative judgment and *I*^2^ for quantitative estimation. We used the best linear unbiased prediction (BLUP) to obtain the overall cumulative exposure-response association of multiple cities. The BLUP allows areas with few daily mortality counts or short series to exploit information from the other areas to share similar characteristics [26]. Finally, we tested the correlation between model variables and mortality through the Wald test.

At last, the minimum mortality temperature, corresponding to the specific temperature associated with the lowest mortality risk. Then we calculated the attributable fraction of cold (below MMT) and heat (above MMT) by deeming the MMT as a baseline reference. Moreover, the continuous temperature of each city was divided into four temperature levels: extreme cold, moderate cold, moderate heat and extreme heat, based on the cutoff values of 2.5th and 97.5th temperature percentiles and MMT referred to previous studies [25,27].

AFs of each city were calculated as follows:

(3) $$AF_{x,t}=1-\exp\left(-\overset{L}{\underset{l=0}{\sum }}\beta_{x_{t,l}}\right)\;$$

(4) $$AN_{x,t}=AF_{x,t}∙\overset{L}{\underset{l=0}{\sum }}\frac{n_{t+l}}{L+1}$$

where $\overset{L}{\underset{l=0}{\sum }}\beta_{x_{t,l}}$ is defined as the cumulative lagged effect of the overall risk of temperature *x_t_* on day *t*; *L* means the maximum lag time (21 days); $n_{t}$ is the number of deaths on day *t*; $AN_{x,t}$ means attribute numbers on day *t*. By summing the $AN_{x,t}$ from all the days in the series, we obtained the total counts of deaths attributable to non-optimum temperatures, then the total attributable fraction was gained by dividing the total number of deaths by the total number of attributable deaths.

### 2.3. Sensitivity Analysis

Considering the difficulty of determining appropriate maximum lag days and degrees of freedom, sensitivity analyses were performed by using alternative maximum lag days (15/25 days) and changing the df of time trends (7–10 df/year). Additionally, we also did sensitivity analysis by controlling for particulate matter 10 μm in diameter (PM_10_) and deaths due to influenza in Tianjin, since pollution and influenza data were available only for Tianjin.

Our study did all analysis with R software (R Development Core Team, 2020) in RStudio (RStudio Team, Boston, MA, USA) with packages mgcv to build GAM, splines and dlnm to create DLNM, mvmeta to do meat regression. All statistical tests were two-sided, and the values with *p* < 0.05 were deemed statistically significant.

## 3. Results

**Figure 1:**
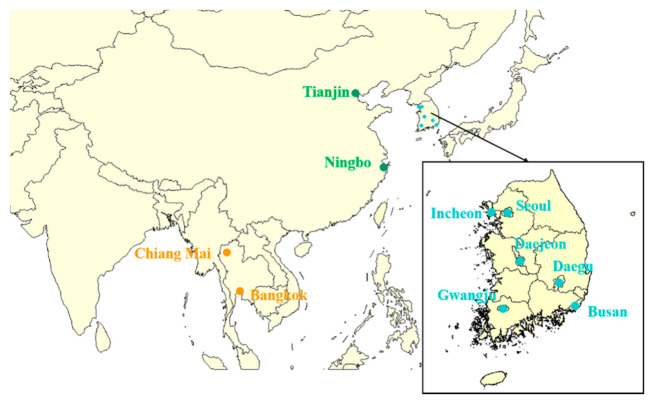
The locations of 10 Asian cities in this study.

Figure 1 gives the location distribution of 10 Asian cities in this study. There are 2 cities in China: Ningbo and Tianjin, 6 cities in Korea: Seoul, Busan, Daegu, Incheon, Gwangju, and Daejeon, 2 cities in Thailand: Bangkok and Chiang Mai. Table 1 shows the descriptive data on total non-accidental deaths, mean apparent temperature and mean relative humidity during the study period in the 10 Asian cities included in this study. The database included 1,416,091 deaths. The total counts for Thailand, Korea and China were found to be 217,470, 888,985 and 374,142, respectively. Generally, the apparent temperature in Thailand (ranging from 10.3 to 42.3 °C) is much higher than in Korea (ranging from −10.8 to 38.4 °C) and China (ranging from −9.9 to 42.1 °C), indicating that climate characteristics varied between the countries.

**Figure 2:**
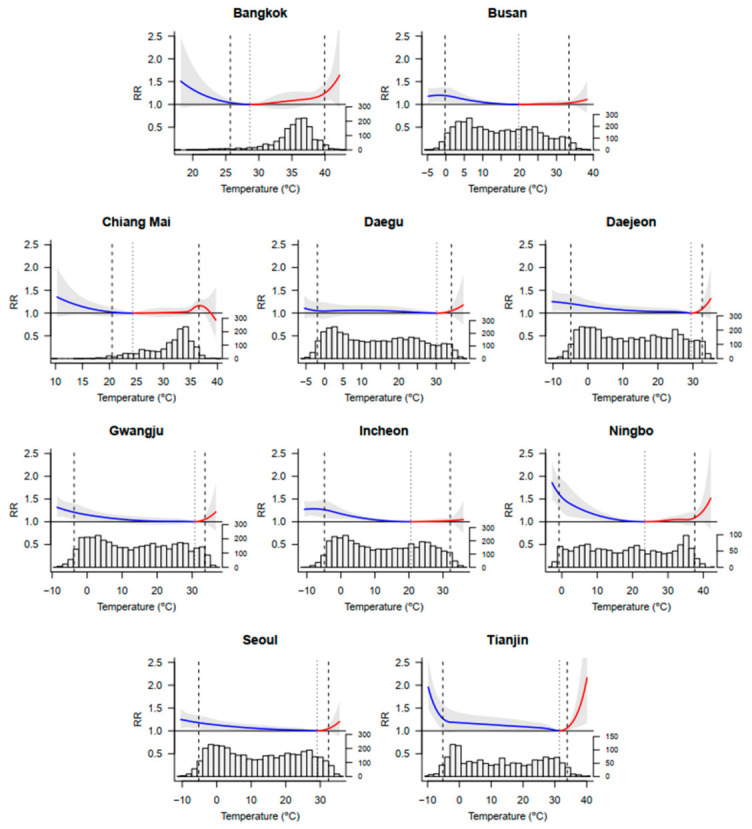
The overall cumulative exposure-response associations in 10 cities. Exposure-response associations as best linear unbiased prediction (with 95% empirical CI, shaded grey) in 10 countries, with related temperature distributions. Gray dotted lines are minimum mortality temperatures, and gray short-dashed lines are the 2.5th and 97.5th percentiles. RR = relative risk.

Figure 2 reveals the cumulative exposure-response curves (estimated by BLUP) and apparent temperature distributions of 10 Asian cities, with the corresponding MMT and the cutoffs to define extreme and moderate temperatures. The histogram plots show that most daily temperatures were lower than the MMT. As shown in Figure 2, the cumulative exposure-response relationship was described by V or L sharp. Both the cold effect (below MMT) and the heat effect (above MMT) were found to be significant for all cities in this study. However, the exposure-response curves of cold and heat are different. Specifically, the curve of the cold effect changes slowly and linearly, while the corresponding heat effect shows a sharp and nonlinear increase, indicating that the cold effect lasted longer and was more delayed than heat. Additionally, although the relative risks (RRs) are highest at extreme cold and extreme hot, relative contributions from moderate cold and hot are higher than extreme, due to the small proportion of extreme temperature days according to the histogram plots.

Table 2 presents the attributable fraction caused by cold and hot temperatures in each city. Overall, the minimum mortality percentile (MMP) in Asia was 79.5th percentile. For cities in Korea and China, the MMP ranged between about the 60th and 90th percentiles of temperature, with the exception of cities in Thailand, where the MMPs were 10th and 5th percentiles. The MMT ranged from 19.71 °C in Busan, Korea, to 31.46 °C in Tianjin, China, and mainly varied between 20 °C and 30 °C. In total, 7.62% of non-accidental mortality was attributed to temperature. However, attributable fraction varied substantially across cities, with the highest estimates in Ningbo (11.78%) and Tianjin (11.54%), China, and the lowest in Chiang Mai (2.84%), Thailand, suggesting that the mortality risk of temperature in China is larger than in the other two countries. Most temperature-attributable deaths were caused by cold temperature, with a fraction of 6.44%, while the hot temperature only accounts for a small part of the burden, with a fraction of 1.18%. This result keeps consistent among cities in Korea and China, while the results in Thailand were the opposite. Sensitivity analysis showed that the overall MMPs and AFs remained almost unchanged when the lag period and df varied and were not dependent on pollution and influenza (Supplementary Table S1).

Table 3 shows the result of the multivariable meta-regression analysis, which included average temperature and temperature variation as meta-predictors. We found that both average temperature and temperature variation showed a significant correlation with non-accidental death counts, with an *I*^2^ of 36.6% and Cochran Q test insignificant, indicating that the residual heterogeneity was low.

## 4. Discussion

In this study, we estimated the association between apparent temperature and non-accidental deaths in ten Asian cities. The exposure-response curves were V or L shaped, with a significant non-accidental mortality increase attributable to cold and hot temperatures, and the cold effect characterized with hysteresis and persistence. The overall MMT was distributed at the 79.5th percentile of temperature, although this percentile varied substantially between cities in different countries. We also found that most temperature-attributable deaths were caused by cold, with a fraction of 6.44%. Generally, the association between temperature and non-accidental mortality is consistent with previous studies [28,29].

Many studies found the relationship between health outcomes and temperature was generally observed as U, V, and J-shaped [9,17,30], similarly, we also found the association between non-accidental mortality and apparent temperature was L or V-shaped. Although the RR corresponding to extreme cold was larger than moderate cold, the moderate cold occurred more frequently and caused more non-accidental deaths. Therefore, the researchers only considering health effect of extreme temperature underestimated the mortality risk of ambient temperature. Global warming has obviously shortened the time of seasonal transformation [31], leading to weather-sensitive diseases such as respiratory diseases due to insufficient time to adapt to the change of seasons. The high number of deaths attributable to moderate cold could be explained by the seasonal transition problem contained in it. Thus, during seasonal transition period, effective measures should be implemented to strengthen people’s awareness of preventing cold for a long time, not only focusing on extreme cold.

There were regional heterogeneities about the mortality risk of temperature in our study. We found more than 10% of non-accidental mortality was attributable to temperature in China, which was much larger than the other two countries, indicating that people living in China are more sensitive to temperature. Gasparrini et al. [9] also found the attributable fraction ranged from 3·37% in Thailand to 11.00% in China. These discrepancies in susceptibility can be explained by a variety of factors, including natural endowment, socioeconomic characteristics, population structure, living habits [32], access to health care, and housing conditions [19]. Therefore, relevant organizations should take relevant adaptive strategies accordingly to deal with the regional specific threat of temperature. Additionally, more temperature-related mortality was caused by heat than cold in Thailand, which is different from Korea and China, and also inconsistent with the results of previous studies in Thailand [33]. This difference might be explained by several reasons. First, the large heat effect in Thailand was attributed to the low MMP (5–10th percentile), while the MMP in China and Korea ranged from about 60th to 90th percentile. A multi-country study also found that the MMPs in tropical areas such as Thailand is lower than other areas [9], in which the MMPs for most countries were at 80th and 90th percentile, with the exception of the tropical areas such as Thailand (60th percentile). This difference is mainly due to the high temperature and relative humidity in Thailand all year. Thailand is located in a tropical region, the daily maximum temperatures are above 30 °C in most regions of Thailand throughout a typical year, without limit to summer [34], thus the weather conditions might differ from China and Korea where cities are situated in the temperate climate zone [35]. Second, under global warming situations, the air temperature in Thailand has had a relatively high increase in recent years [36]. Studies on future temperature-related health risk found an increase in heat-related mortality and a decline in cold-related mortality [5,37,38]. For instance, Ballester et al. reported that the rise in deaths from heat will compensate the reduction of cold-related mortality during the second half of the century [37]. The MMTs in Australia, Brazil and the United Kingdom showed a decreasing trend between around 1990 and around 2010, from 17.2–27.4 °C in the first year to 16.3–24.8 °C in the last year [39], indicating that the mortality attribute to cold would decline with time. As a tropical developing country, Thailand is more sensitive to climate warming due to the limited public health infrastructure and adaptive capacity [35]. Therefore, the temperature-attributable mortality caused by cold may be overweighted by heat during our study period in Thailand (2013–2016), whereas previous studies, in which more temperature-attributable deaths were caused by cold (2.61%) than by heat (0.76%) in Thailand, focused on an earlier time (1999–2008) [9]. Considering the regional heterogeneity, regional specific policy should be planned to minimize the health risk of temperature.

Our result showed that compared with heat, cold temperature plays an important role in non-accidental mortality overall, although both heat and cold contribute to mortality. These different effects of hot and cold temperatures on human health can be explained by several potential mechanisms. First, the underlying physiopathological mechanisms that link heat and mortality is not completely clear. Exposure to heat may cause heart rate increase, heart overload and blood viscosity increase, which could contribute to water-electrolyte imbalance [40]. For exposure to cold temperature, by exciting the human sympathetic nerves, cold is associated with a variety of autonomic responses in humans, including vasoconstriction, increased blood pressure and heart rate [41]. These changes may increase the mortality rate of patients with cardiovascular and cerebrovascular diseases. In addition, cold temperature has also been found to diminish the movement of human bronchial cilia and the phagocytosis of alveolar macrophages, which weakens the airway defense ability, increasing the mortality rate of respiratory diseases [42]. These biological mechanisms of cold effect persist for longer and are more severe than the effects of hot temperature. Second, people would use air conditioners and fans to adjust the indoor temperature and consciously reduce outdoor activities during a hot summer to decrease the heat effect. Heating systems were available only in some areas of Asia, with a low awareness of preventing cold, leading to a larger effect than heat.

According to the result of multivariate meta-analysis, we observed that the daily average temperature and temperature variability were related with mortality significantly, consistent with previous studies [9,43,44]. For instance, a multi-city study conducted by Yang et al. in China showed that 17.1% of CVD deaths were attributable to daily mean temperature [17]. Another study reported that a 1 °C increase in temperature variability was significantly associated with a 0.9% increase in all-cause mortality [43]. The mortality risk of temperature change could be explained by physiology and immunology evidence [43]. Temperature change might affect humoral or cellular immunity [45] and result in a mild inflammatory reaction, which results in respiratory diseases such as chronic obstructive pulmonary disease [46]. Moreover, the inefficient thermoregulatory response to sudden temperature change may also lead to a burden of cardiovascular-related markers such as heart rate, heart rate variability, blood pressure [43], blood platelets, red cells, and viscosity [45,47], which may trigger circulatory related diseases.

Though existing studies suggest that influenza, temperature, and air pollution may have a synergistic effect on mortality, and they are often defined as each other’s confounders [48], whether pollution and influenza are confounders of temperature remains under discussion [49,50]. In this study, we found that the mortality burden attribute to temperature in Tianjin remained robust after adjusting PM_10_ and influenza deaths, suggesting that air pollution levels and influenza epidemics may not influence the mortality risk of temperature.

This study has several strengths. First, we selected apparent temperature as the temperature exposure indicator. Many previous temperature-related studies have used a single temperature metric (e.g., daily mean/minimum/maximum temperature) to characterize the temperature, which may not measure human exposure to ambient temperature very well, since human body temperature is related to many weather variables. Humans regulate body temperature by sweating, which is affected by humidity. Many methods were developed to diagnose heat stress synthetically, especially moist thermodynamical quantities, such as the Wet Bulb Globe Temperature (WBGT) and Heat Index (HI), but there was no uniform way to calculate these metrics [51]. As a comprehensive biometeorological index of ambient temperature and relative humidity, AT describes the perception of temperature and thermal stress better than air temperature [52]. Additionally, compared with other temperature indicators, Zhang et al. considered apparent temperature to be the most important predictor for all-cause mortality [53]. Second, this research quantitatively assessed the overall attributable fraction of temperature exposure rather than only analyzing the relative risk of temperature. We revealed that the mortality effect of moderate temperature was the largest among moderate and extreme temperatures. Our result provides evidence for further studies to explore the weather-sensitive diseases.

Limitations must be acknowledged. First, we failed to conduct subgroup analysis by gender or age to explore the sensitive population because of the limitation of information. Second, given the huge geographic regions, different climatic characteristics in Asia, ten cities of three countries included in this study can not represent Asia as a whole. Further studies are needed to overcome these limitations.

## 5. Conclusions

In summary, our study suggested that apparent temperature was associated with an increase in non-accidental mortality. Most of the temperature-related mortality burden was attributable to cold. This study provided evidence of the mortality risk of temperature, including moderate and extreme. Our result had important significance for planning public health interventions to minimize the health effects of adverse temperatures.

## Figures and Tables

**Table 1:** Descriptive analysis of non-accidental daily death counts and meteorological variables in 10 Asian cities.

Country	City	Study Period	Death Counts	Apparent Temperature (°C) (Range)	Relative Humidity (%) (Range)
Thailand	Chiang Mai	2013–2016	34,195	31.0 (10.3–39.7)	69.4 (38.9–95.5)
Thailand	Bangkok	2013–2016	183,275	35.2 (18.2–42.3)	74.2 (46.0–97.0)
Thailand	- *	2013–2016	217,470	33.1 (10.3–42.3)	71.8 (38.9–97.0)
Korea	Seoul	2000–2010	375,082	12.6 (−10.4–35.7)	61.8 (19.4–96.5)
Korea	Busan	2000–2010	183,275	14.9 (−4.8–38.4)	62.7 (16.2–99.0)
Korea	Daegu	2000–2010	111,032	14.3 (−5.4–37.3)	57.5 (16.9–95.9)
Korea	Incheon	2000–2010	106,282	12.5 (−10.8–36.1)	67.3 (26.0–98.4)
Korea	Gwangju	2000–2010	57,216	13.9 (−8.7–36.9)	66.6 (19.6–98.1)
Korea	Daejeon	2000–2010	56,098	12.8 (−10.4–35.2)	65.7 (24.2–97.6)
Korea	-	2000–2010	888,985	13.5 (−10.8–38.4)	63.6 (16.2–99.0)
China	Tianjin	2008–2011	245,847	17.0 (−9.9–39.0)	72.1 (15.0–97.0)
China	Ningbo	2008–2011	128,295	19.2 (−2.8–42.1)	72.2 (19.0–95.0)
China	-	2008–2011	374,142	15.7 (−9.9–42.1)	64.4 (15.0–97.0)
Total			1,416,091	15.6 (−10.8–42.3)	64.5 (15.0–99.0)

* - means taking the country as a whole.

**Table 2:** Attribution risk of the impact of short-term temperature exposure on non-accidental mortality in ten Asian cities.

Country	City	Minimum Mortality Percentile	Minimum Mortality Temperature (°C)	Total (%) (95% CI)	Cold (%) (95% CI)	Heat (%) (95% CI)
Thailand	Chiang Mai	10	24.32	2.84 (−3.89, 8.83)	0.17 (−0.40, 0.71)	2.67 (−4.51, 9.09)
Thailand	Bangkok	5	28.66	8.91 (−3.82, 19.78)	0.31 (−0.06, 0.67)	8.61 (−3.74, 19.45)
Korea	Seoul	91	29.12	6.46 (0.80, 11.11)	6.18 (0.98, 11.19)	0.28 (−0.15, 0.68)
Korea	Busan	65	19.71	5.13 (0.55, 8.79)	4.74 (1.12, 8.34)	0.39 (−0.70, 1.44)
Korea	Daegu	90	30.17	3.96 (−3.56, 10.42)	3.68 (−4.15, 10.67)	0.28 (−0.33, 0.81)
Korea	Incheon	66	20.56	6.77 (2.91, 10.16)	6.48 (2.60, 9.74)	0.29 (−0.62, 1.34)
Korea	Gwangju	91	30.74	6.05 (0.15, 11.66)	5.75 (−0.85, 11.10)	0.30 (−0.21, 0.78)
Korea	Daejeon	91	29.45	7.62 (0.67, 13.92)	7.15 (0.01,13.03)	0.46 (−0.21, 1.03)
China	Tianjin	93	31.46	11.54 (1.12, 20.38)	11.08 (0.37, 19.60)	0.46 (0.13, 0.72)
China	Ningbo	63	23.48	11.78 (4.62, 18.12)	10.48 (4.28, 15.68)	1.30 (−0.92, 3.35)
Asia		79.5	-	7.62 (4.95, 9.83)	6.44 (3.97, 8.63)	1.18 (0.14, 2.15)

**Table 3:** Second-stage random-effects meta-regression models.

Model	Predictor	Test for Predictor	Q Test	*I^2^* Statistic
random-effects meta-regression	Average temperature	0.0316	0.0162	36.6%
	Temperature variation	0.0348		

## Data Availability

Restrictions apply to the availability of these data. Data was obtained from Center for Disease Control and Prevention of Ningbo and TianJin, China Meteorological Data Sharing Service System, Dr. Ho Kim, Thailand Meteorological Department, Ministry of Digital Economy and Society, Strategy and planning division, Ministry of public health, and are available with the permission of them.

## Supplementary Materials

The following are available online at https://www.mdpi.com/article/10.3390/ijerph18094675/s1, Table S1: Computed on the attributable fraction (%) to temperature (total, heat, and cold components), by varying lag, df, and controlling air pollution and influenza.
