# Supplementary material for: The Mortality Effect of Apparent Temperature: A Multi-City Study in Asia
Source: Int J Environ Res Public Health. 2021 Apr 28;18(9):4675. doi: 10.3390/ijerph18094675 (PMC8124769; doi:10.3390/ijerph18094675)
Supplement: Supplementary file 1 [file ijerph-18-04675-s001.zip › ijerph-1158946-supplementary.pdf]

Table S1. Computed on the attributable fraction (%) to temperature (total, heat, and cold components), by varying lag, df, and controlling air pollution and influenza.

Modelling choices	Minimum mortality percentile	Total(%) (95% CI)	Cold(%) (95% CI)	Heat(%) (95% CI)
**Lag period: 15 days (10 cities)**	85	7.63 (5.30, 9.78)	6.75 (4.61, 8.70)	0.88 (-0.09, 1.67)
**Lag period :25 days (10 cities)**	58	6.09 (1.73, 9.63)	3.48 (1.49, 5.25)	2.60 (-1.78, 5.90)
**Df/year for seasonal control: 7 (10 cities)**	81	6.45 (4.07, 8.64)	5.38 (3.00, 7.28)	1.08 (0.12, 1.95)
**Df/year for seasonal control: 9 (10 cities)**	83	8.43 (5.57, 11.19)	7.29 (4.32, 9.72)	1.15 (0.02, 2.09)
**Df/year for seasonal control: 10 (10 cities)**	91.5	8.24 (5.75, 10.44)	7.29 (4.85, 9.62)	0.94 (-0.06, 1.86)
**Air pollution control (Tianjin)**	93	13.49 (2.54, 22.27)	13.05 (2.68, 21.96)	0.44 (0.13, 0.71)
**Influenza control (Tianjin)**	93	11.74 (1.49, 20.46)	11.28 (0.85, 20.06)	0.46 (0.12, 0.73)
**Air pollution+influenza control (Tianjin)**	93	13.73 (2.85, 22.86)	13.27 (3.21, 22.19)	0.45 (0.15, 0.70)
